# Adaptive training algorithm for robot-assisted upper-arm rehabilitation, applicable to individualised and therapeutic human-robot interaction

**DOI:** 10.1186/1743-0003-10-102

**Published:** 2013-09-28

**Authors:** Radhika Chemuturi, Farshid Amirabdollahian, Kerstin Dautenhahn

**Affiliations:** 1 Adaptive Systems Research Group, Department of Computer Science, University of Hertfordshire, College Lane campus, Hatfield AL10 9AB, UK

**Keywords:** Stroke, Upper-arm rehabilitation, Lead-lag roles, Adaptable system, Embedded-virtual environments, Reach-return movements

## Abstract

**Background:**

Rehabilitation robotics is progressing towards developing robots that can be used as advanced tools to augment the role of a therapist. These robots are capable of not only offering more frequent and more accessible therapies but also providing new insights into treatment effectiveness based on their ability to measure interaction parameters. A requirement for having more advanced therapies is to identify how robots can 'adapt’ to each individual’s needs at different stages of recovery. Hence, our research focused on developing an adaptive interface for the GENTLE/A rehabilitation system. The interface was based on a lead-lag performance model utilising the interaction between the human and the robot. The goal of the present study was to test the adaptability of the GENTLE/A system to the performance of the user.

**Methods:**

Point-to-point movements were executed using the HapticMaster (HM) robotic arm, the main component of the GENTLE/A rehabilitation system. The points were displayed as balls on the screen and some of the points also had a real object, providing a test-bed for the human-robot interaction (HRI) experiment. The HM was operated in various modes to test the adaptability of the GENTLE/A system based on the leading/lagging performance of the user. Thirty-two healthy participants took part in the experiment comprising of a training phase followed by the actual-performance phase.

**Results:**

The leading or lagging role of the participant could be used successfully to adjust the duration required by that participant to execute point-to-point movements, in various modes of robot operation and under various conditions. The adaptability of the GENTLE/A system was clearly evident from the durations recorded. The regression results showed that the participants required lower execution times with the help from a real object when compared to just a virtual object. The 'reaching away’ movements were longer to execute when compared to the 'returning towards’ movements irrespective of the influence of the gravity on the direction of the movement.

**Conclusions:**

The GENTLE/A system was able to adapt so that the duration required to execute point-to-point movement was according to the leading or lagging performance of the user with respect to the robot. This adaptability could be useful in the clinical settings when stroke subjects interact with the system and could also serve as an assessment parameter across various interaction sessions. As the system adapts to user input, and as the task becomes easier through practice, the robot would auto-tune for more demanding and challenging interactions. The improvement in performance of the participants in an embedded environment when compared to a virtual environment also shows promise for clinical applicability, to be tested in due time. Studying the physiology of upper arm to understand the muscle groups involved, and their influence on various movements executed during this study forms a key part of our future work.

## Background

According to the World Health Organisation’s (WHO) statistics [1] the average life expectancy of the world’s population is increasing consistently. With an increasingly ageing population, the burden of disease on the economies of many countries increases. Stroke, being one of the leading causes of disabilities in many countries, is leaving a considerably large number of people to live with its consequences. The incidence of stroke increases with age and estimates show there will be a marked increase in the number of stroke events in EU countries from approximately 1.1 million per year in 2000 to 1.5 million per year in 2025 [2].

Rehabilitation is the process by which patients with strokes undergo treatment to help them return to normal life as much as possible by regaining and relearning the skills of everyday living [3]. It also aims at helping the survivor to understand and adapt to difficulties, prevent secondary complications and educate family members to play a supporting role. Early intervention is believed to be effective on the quality of rehabilitation [4]. Stroke sufferers with functional impairments often do not reach their full potential for recovery when discharged from inpatient settings [5-7]. The major hurdle in offering rehabilitation to stroke sufferers is the lack of sufficiently trained personnel. One of the potential solutions could be providing the existing personnel with advanced tools that can reduce the role of the therapist without any compromise on the treatment effectiveness.

The scope of stroke recovery spans very wide, recovery can start as early as in the sub-acute stage (immediately after the incidence of stroke) and can extend into the chronic stages too (six months post stroke) [8]. Recovery is largely variable between patients in every stage and hence it is necessary that the rehabilitation techniques need to be geared towards patients’ specific motor deficits. Reviews of previous post-stroke rehabilitation studies [9-11], involving a robotic-assistance, suggested that robotic therapy will have the greatest impact if it can reduce the role of the therapist without the loss of treatment effectiveness and can motivate the patients to exercise independently. This reiterates the need for robotic therapy to be highly adaptable according to the specific needs and performance of the patient.

Robots also have the capability to track many interaction parameters; this feature could be used to continuously track the performance of the patient during therapy sessions. Studies [12,13] also suggest that robot-aided training will be effective if it is progressive and challenging according to the patient’s ability. In order to achieve this, the performance of the patient has to be tracked and the training has to be altered accordingly.

The goal of our research with the GENTLE/A rehabilitation system is to identify the parameters that can inform the contribution of the participant during a human-robot interaction (HRI) session and adapt the assistance/resistance offered by the system based on the participant’s contribution. Previous studies [14,15] conducted with the GENTLE/A rehabilitation system demonstrated that the leading-lagging performance of the participant could be identified using the positional coordinates. Robots often use a reference trajectory model to guide the movement of patients. The error between the robot recorded coordinates and the reference trajectory coordinates at a given time was used to identify the lead-lag contribution of the participant interacting with the system.

The results [14] with single axis or planar (horizontal XY plane) point-to-point movements during a preliminary study showed that the sign of the error was impacted by the type of movement (reaching away or returning towards the body) and also by the introduction of elevation into the experimental workspace. These findings were further explored in a 3-dimensional workspace in our next study, during which scenarios were created where the participants were asked to intentionally lead/lag the interaction using feedback provided by the graphical user interface, while the robot was programmed to follow a human arm modelled trajectory.

Our results [15] showed that vector projections of position data recorded could inform about the lead-lag role of the participant. It was observed that participants were not always successful in leading the interaction during the leading scenario. Participants could lead the interaction during the 'reaching’ (moving away from the body), but could not always lead the interaction during the 'returning’ (returning towards the body). With the set of points that were chosen for these previous studies, all the reaching movements were against gravity and all the returning movements were towards gravity. Investigations into reasons underlying our observations led to some interesting conclusions listed in Table 1. Table 1 also lists the aims of the current study based on these conclusions. We proposed an adaptive algorithm that would tune the duration given to execute point-to-point movements according to the leading-lagging role of the interacting participant. This paper presents the algorithm proposed to adapt the GENTLE/A rehabilitation system according to the user’s ability, and the results from the study aimed to evaluate the adaptability of the system. The influence of various input parameters on the adaptive nature of the system is assessed using the regression model and its applicability in clinical settings is discussed.

**Table 1:** Design and aims of current study

***Conclusions from previous studies with the GENTLE/A rehabilitation system ***[14,15]	***Design and aims of current study***
Duration given to execute a point-to-point movement was either too short and did not allow the participant to lead the interaction or too long and led to a lazy performance of the participant.	*Algorithm:* An algorithm was proposed that would adapt the duration given to execute a point-to-point movement to reach an optimum value according to the performance of the participant.
Type of the movement (reaching/returning) was influencing the performance of the participant.	*Points on cube:* Set of points was chosen such that different combinations of reach-return and ground level - against gravity – towards gravity movements were executed during the experiment.
Direction of movement (away/towards) with respect to gravity was influencing the performance, as the participant’s arm was not gravity compensated.	
Perception of depth in the Virtual Reality (VR) environment presented during the previous studies was felt difficult by the participants.	*Embedded vs. Virtual:* The virtual environment has been modified to improve the depth perception and an embedded set-up was also introduced to compare the performance of the participants in embedded vs. virtual environments.

## Methods

### Participants

Thirty-two healthy participants took part in the experiment, age range 33.6 ± 9.4 (mean ± standard deviation), including 18 female and 14 male participants. Detailed demographics of the participants are provided in Table 2. Written informed consent was obtained from every participant before inclusion in the studies and ethical approval of the evaluation protocol was obtained from the Ethics committee of University of Hertfordshire (under approval number 1112/45). Data from two participants (ParticipantID 28 and 29) remained fluctuating throughout the experiment, possibly due to the participants’ inability to master how to perform the task at hand. Hence the data recorded from these two participants was excluded from analysis. The data collected from thirty participants (*n* = 30) was used for the analysis purposes.

**Table 2:** Participant demographics

***Participant ID***	***Age***	***Gender***	***Dominant hand***	***Vision correction***
1	25	F	R	Y
2	24	F	R	N
3	32	F	R	Y
4	47	F	R	N
5	32	M	R	N
6	23	M	R	Y
7	40	M	R	N
8	27	M	R	Y
9	29	F	R	Y
10	29	M	R	Y
11	50	F	R	Y
12	33	F	R	Y
13	32	F	R	N
14	25	F	R	Y
15	39	M	R	N
16	26	M	R	Y
17	29	M	R	N
18	31	M	R	Y
19	32	F	R	Y
20	30	F	R	Y
21	26	F	R	N
22	27	F	R	Y
23	32	M	R	Y
24	60	F	R	Y
25	45	M	R	N
26	42	F	R	N
27	29	M	R	N
28	36	M	R	N
29	22	M	R	N
30	53	F	R	Y
31	24	F	R	Y
32	44	F	R	N

F – Female, M – Male.

L – Left hand, R – Right hand.

Y – Yes (wears glasses or contact lens), N – No.

### Experimental set-up

**Figure 1:**
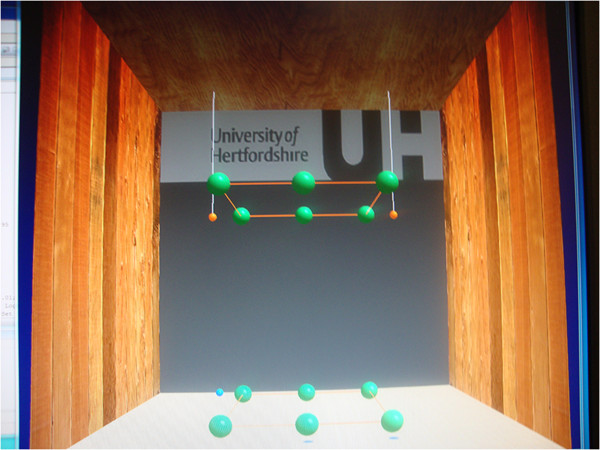
**Embedded set-up.** Ping-Pong balls and stickers on the table-mat served as the real objects alongside the virtual targets represented as green balls on the monitor.

The robotic component of the GENTLE/A rehabilitation system, the HapticMaster (HM) [16] was programmed to follow the Minimum Jerk Trajectory (MJT) [17] that mimics the human arm movement with similar smoothness. HM can be operated in various modes, patient-passive, active-assisted and patient-active, which were originally designed to work on the GENTLE/S system [18,19]. The Virtual Reality (VR) environment was created using OpenGL. The ring end-effector used for arm connection in clinical settings was replaced with a ball end-effector as healthy participants did not require additional arm support. The participants were asked to hold the ball attached to the end of robotic arm (see Figure 1) with their dominant hand and move between various points displayed on the monitor. The path between a source and a target point was called a 'segment’.

#### Points on cube

**Figure 2:**
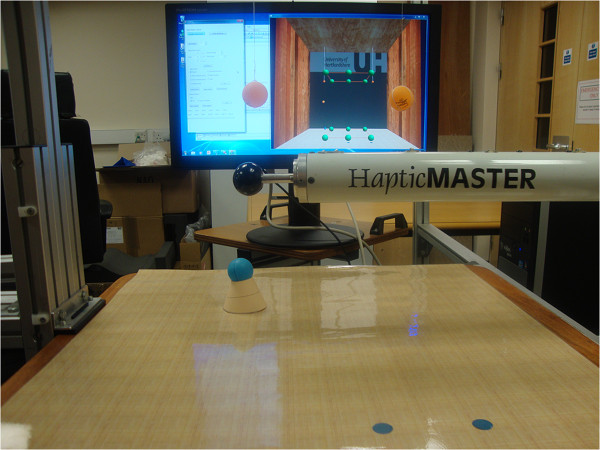
**Virtual environment set-up.** Green balls represented source and target points for various segments. An inflating target ball plus an audio cue was used to start movement for each segment. Red and grey cylinders, shown in Figure 3, appeared as the movement progressed from source to target.

Figure 2 shows the VR set-up with cube and balls. The green balls represented the source and target points for various segments. The cube was formed such that points on the cube facilitate different combinations of movements including reach-return, ground level-against gravity-towards gravity, etc.

#### Embedded vs. Virtual

Previous research in our group tested the performance of participants in environments with different levels of realism [20]. Results showed that participants performed better in an embedded reality setting when compared to a purely virtual setting. In order to facilitate the comparison of the participants’ performance in the presence/absence of an embedded object, an embedded reality set-up was created. This is also with the aim to reduce the cognitive load needed to perceive 3D information on a 2D display. Figure 1 shows both the embedded and the virtual targets. Help from an embedded object was provided for some points and other points only had a virtual representation. Ping-Pong balls were either hung from the top frame or placed on the table-mat at a small elevation, in close proximity to the virtual balls of the cube, to provide assistance for depth perception. With respect to the points located on the front face of the cube that were closer to the participant, visible stickers were placed on the table-mat, just below the positions where the actual points of the cube exist in the workspace.

#### Modes of operation

The HM was programmed to operate in two modes for the purpose of this experiment.

1. Passive: Participant passive – robot active

In the passive mode the Cartesian coordinates of the source and target points along with a set duration are used by the HM to transition from the source to the target points following the MJT. The HM can execute the entire segment while the participant remains passive.

2. Active-assisted: Participant and robot work together

During the active-assisted mode the participant has to initiate the movement towards the target point and once the movement starts progressing towards the target, the HM becomes active and the robot and the user work in coordination to reach the target.

These modes used the same algorithm as those implemented for the GENTLE/S rehabilitation system [19] to allow future comparison between the findings in both studies. In order to test the lead-lag contribution of a participant during a human-robot interaction session, the passive and the active-assisted modes were chosen as robot was often active during these modes.

#### Lead-Lag scenarios

Lagging performance: During the passive mode the participant was asked to remain passive and the robot was programmed to execute the activity. During the execution of a segment, at the beginning of every sampling interval, the MJT position was computed (given start and end positions, as well a guide duration using the algorithm given in [17]) and then the robot gently pulled the participant’s arm to catch up with the MJT position. Given the instructions to remain passive, the passive mode was chosen for studying the 'lagging’ performance of the participant.

**Figure 3:**
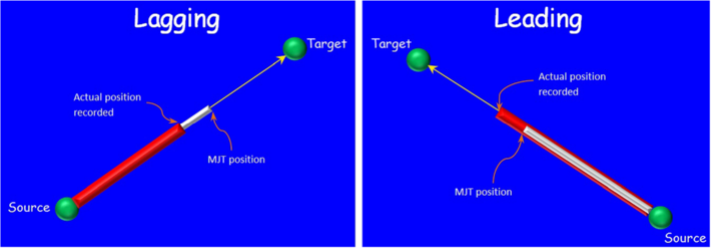
**Leading and Lagging scenarios.** Grey cylinders represent the path to be followed by the robot according to MJT. Red cylinders represent the actual path achieved by the robot while the participant was interacting with the system.

Leading performance: In the active-assisted mode the participant had to initiate the activity and subsequently the participant and the robot could work in coordination to finish the activity. The HM was programmed to follow the MJT. This was the first run of the active-assisted mode and was termed as AA1. In the second run of the active-assisted mode (AA2), the participant was encouraged to use additional force to pull the robot arm to reach the target point quicker than the set duration. Hence AA2 was considered for studying the 'leading’ performance of the participant. Figure 3 shows a pictorial representation of lagging and leading scenarios which was used to provide feedback to participants during their lagging and leading performances.

### Procedures

**Figure 4:**
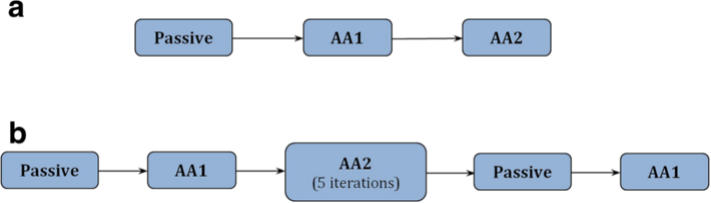
**Flow-chart style representation of experimental procedure.** The training phase **(a)** was followed by the actual-performance phase **(b)** during the experimental session.

The experiment was conducted in two phases: (a) Training, (b) Actual-Performance. Figure 4 shows a flow-chart style representation of the experimental protocol. During both phases, participants held the gimbal (see black ball in Figure 1) to follow or lead the robot in its trajectory.

(a) * Training:* In the training phase each mode (passive, AA1 and AA2) was executed at least once or a few times until the participant became familiar with the operation.

(b) * Actual-performance:* The participants executed the passive and the AA1 modes twice, at the beginning and then at the end of the actual-performance phase. The AA2 mode was executed five times during which the system attempted to adapt according to the algorithm implemented that used the interaction parameters recorded. The participants executed thirteen segments in every mode following the same sequential order.

### Performance measures

Every participant took part in one experimental session. During the session the robot recorded the position, velocity and force sensed at the end-effector in 3D Cartesian space. The duration to execute a segment was recorded for every segment and the following parameters were computed.

Tau (τ): Tau (τ) was calculated using the start and end time of the trajectory segment. The main purpose of τ was to map the segment execution time to a parameter between -1 and 1 for making the comparison easier.

*Effort*_*Actual*_: *Effort*_*Actual*_ was calculated as the projection of the vector achieved by the participant onto the straight-line vector joining the source and target points of the trajectory segment.

*Effort*_*MJT*_: Similarly *Effort*_*MJT*_ was calculated from the MJT vector and the straight line vector joining the source and target points.

Δ*Effort*: *Effort*_*MJT*_ gave the contribution of the robot without any assumed contribution from the participant and *Effort*_*Actual*_ gave the combined contribution of participant and robot. Hence a difference between these two parameters and the sign of Δ*Effort* indicated the leading/lagging performance of the participant.

Σ(Δ*Effort*): Summation of all the Δ*Effort* samples from source to target of a segment was calculated as Σ(Δ*Effort*) and this parameter indicated whether the participant’s performance was leading/lagging for major part of the segment.

A more detailed explanation about the parameters computed can be found in our previous publications [14,15].

### Proposed adaptive algorithm

As Σ(Δ*Effort*) was the parameter indicative of the lead-lag role of the participant [15], the contribution of the participant during any interaction session was assumed to be proportional to this parameter. The algorithm below uses Σ(Δ*Effort*) as a performance indicator and adjusts the duration given to execute a segment through repetitions in the AA2 mode, to reach an optimal value.

$$\begin{array}{c} \mathrm{if}\;\overset{n}{\underset{i=1}{\sum }}\Delta \mathrm{Effor}t_{\mathrm{Seg}-k}>0\;/*\mathrm{participant}\;\mathrm{lagging}*/\\ \left(\mathrm{duratio}n_{\mathrm{Seg}-k}+\delta \right)\;\\ \mathrm{else}/*\mathrm{participant}\;\mathrm{leading}*/\\ \begin{array}{c} \left(\mathrm{duratio}n_{\mathrm{Seg}-k}-\delta \right)\;\\ \mathrm{where}\;\delta \propto \overset{n}{\underset{i=1}{\sum }}\Delta \mathrm{Effor}t_{\mathrm{Seg}-k}\;\mathrm{and}\;\delta \;\epsilon \;\left[0.0,1.0\right]\;\\ n-\mathrm{number}\;\mathrm{of}\;\mathrm{samples}\;\mathrm{recorded}\;\mathrm{during}\;\mathrm{segment}-k \end{array} \end{array}$$

This algorithm in effect increases the duration to execute a segment by a small amount (*δ*), that is proportional to the Σ(Δ*Effort*) which is calculated from the recorded interaction parameters, in case where the participant is lagging the reference trajectory. Similarly the duration is reduced by a small amount where the participant is leading the interaction.

## Results

The main aim of this study was to test the adaptability of the GENTLE/A system to tune the duration to execute a point-to-point movement. Therefore the first step of data analysis involved studying the pattern in which the duration for each segment varied through repetitions of the AA2 mode. During the experiment the participants executed thirteen segments traversing between different points presented in Figure 2.

The thirteen segments were executed in the same sequential order during each mode, for all the participants. A segment starting at a source *point k* and ending at a target *point k + 1* was referred to as *seg-k* through the data analysis. Table 3 demonstrates the pattern in which the segment duration varied for one of the participants (Participant 2) during the experiment.

**Table 3:** Adaptation of segment duration (seconds) in the actual-performance phase for Participant 2

***ID***	***Mode***	***Seg-1***	***Seg-2***	***Seg-3***	***Seg-4***	***Seg-5***	***Seg-6***	***Seg-7***	***Seg-8***	***Seg-9***	***Seg-10***	***Seg-11***	***Seg-12***	***Seg-13***
2	AA1	4	4	4	4	4	4	4	4	4	4	4	4	4
2	AA2	3.4	3	3	3	3.6	3.8	3	3.8	3.4	3	3	3.6	3
2	AA2	2.4	2	2.2	2	2.6	2.8	2	2.8	2.6	2.8	1.8	2.6	2
2	AA2	2.2	**1.6**	1.8	1.6	**2.4**	1.8	1.8	1.8	2.2	2.8	1.6	2.2	**1.6**
2	AA2	1.6	**1.6**	**1.6**	1.2	**2.4**	**1.6**	**1.6**	**1.6**	2	**2.6**	1.2	**2**	**1.6**
2	AA2	1.8	**1.6**	**1.6**	1	**2.4**	**1.6**	**1.6**	**1.6**	1.8	**2.6**	1	**2**	**1.6**

Figures in bold highlight the durations that remained constant for two or more repetitions of the AA2 mode.

### Constant optimum duration rule

If the duration remained constant for two or more iterations without a further change as the iterations progressed, we considered the duration to have reached a constant optimum value for that segment.

Applying the above rule, it can be observed from Table 3 that nine out of thirteen segments reached a constant optimum duration within five iterations of the AA2 mode for Participant 2. The Table A (presented as an Additional file 1) gives the pattern change of duration during the five iterations of the AA2 mode of all the participants of the study. In general for all the participants and during all the segments, the duration always scaled down from the default duration set at the beginning of the five iterations. This pattern change in the duration was studied from two viewpoints,

1) *Iteration level:* The variations in the number of iterations required in reaching a constant optimum value of duration from participant to participant.

2) *Segment level:* The variations in the number of participants reaching a constant optimum duration for different segments.

### Iteration level analysis

**Figure 5:**
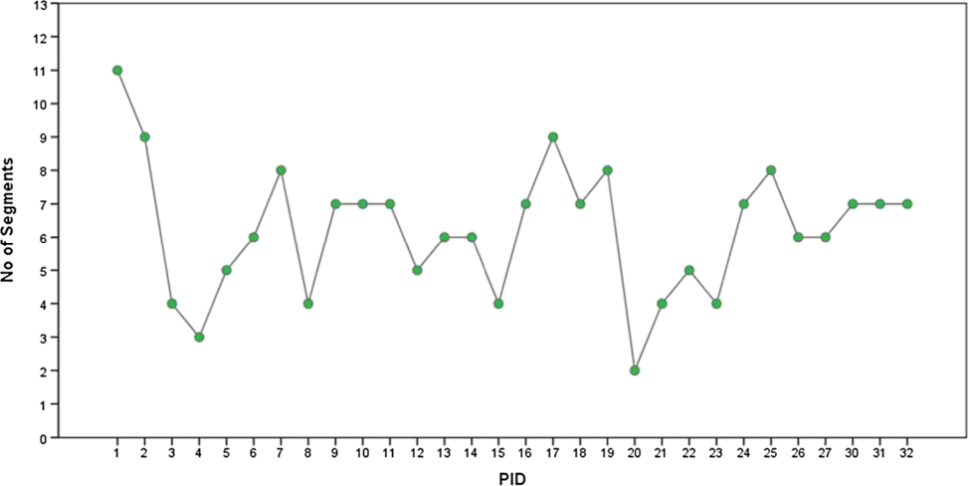
**Participant vs No. of Segments.** Line chart representation of number of segments that reached constant optimum value of duration for every participant within the five iterations of the AA2 mode.

The first observation while studying the pattern change of duration was the number of segments that reached a constant optimum duration within the five iterations of the AA2 mode for each participant. Figure 5 shows that the number of segments that reached a constant optimum value of duration within five iterations of the AA2 mode varied across participants. It could also be observed that for some of the participants, few of the segments reached a constant value within the first 2-3 iterations, and entered a varying duration phase again in the later iterations. The best possible explanation for this change could be that participants aimed to outperform the robot during the later iterations. During the AA2 mode, which is the testing condition for the leading scenario, the participants were asked to use additional force to lead the robot, so although the participants reached their comfortable duration in the first 2-3 iterations, they tried to push themselves harder to outperform the robot and this could have led to further changes in the duration.

### Segment level analysis

**Figure 6:**
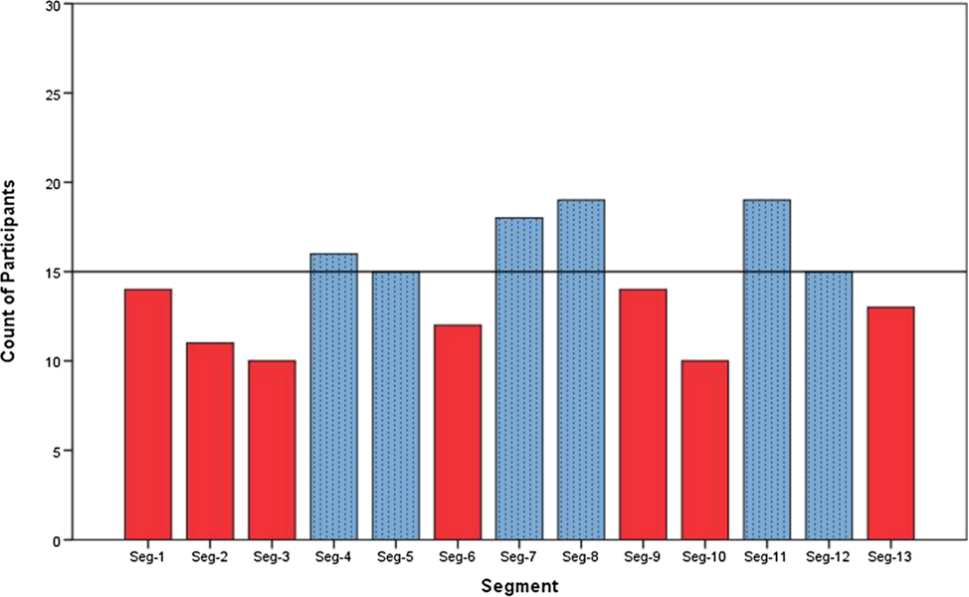
**Segment vs No. of Participants.** Bar chart representation of number participants reaching a constant optimum value of duration during each segment within the five iterations of the AA2 mode.

The second observation concerned varying number of participants reaching a constant optimum value for duration during different segments. It can be observed from Figure 6 that for segments 8 and 11, nineteen out of thirty participants reached a constant optimum value of duration within five iterations. During segment 7 the count was (18/30), segment 4 - (16/30) and segments 5 and 12 - (15/30). For the rest of the segments (bars in’red’) less than half of the participants reached a constant optimum value of duration. This led to the investigation of any underlying patterns in the execution of various segments.

The key observation of pattern change in durations during the five repetitions of the AA2 mode was, the default duration set at the beginning of the first repetition almost always scaled down during all the segments for all the participants by the end of the five repetitions. The patterns observed at iteration level and segment level inform that the number of repetitions required to reach a constant optimum value of duration should be personalised. In addition the patterns observed at segment level led to the investigation of any underlying patterns in the execution of various segments and forms the major part of the results and analysis.

The thirteen segments executed by the participants varied in length. Among these segments, few segments had an embedded object closer to the target point in addition to the target point displayed as a green ball on the monitor as a virtual object. Some segments required a reaching movement (moving away from the body) and some required a returning movement (moving towards the body). The first four segments were executed at ground level without the requirement to move against or towards gravity, while the rest of the segments either moved towards ground or away from it.

**Figure 7:**
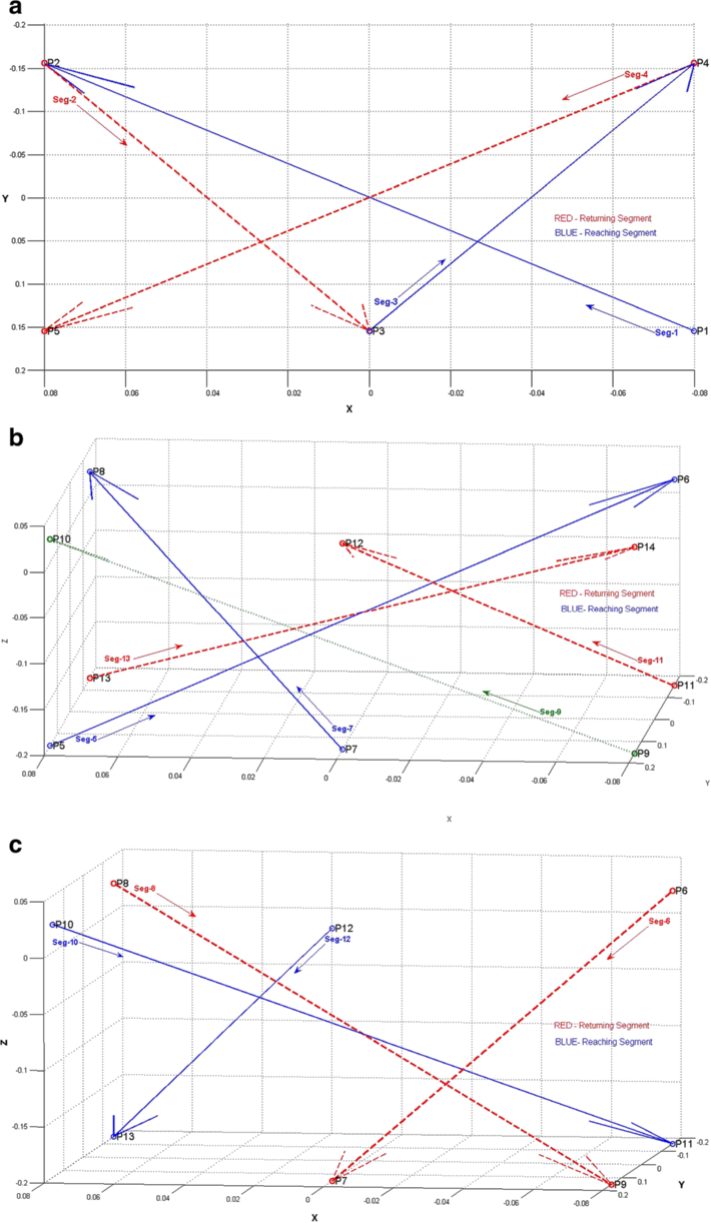
**Pictorial representation of the thirteen segments executed during each mode. (a)** Segments (Ground Level). Segments 1, 2, 3 and 4 executed at ground level (horizontal XY plane) without the influence of gravity. **(b)** Segments (Against Gravity). Segments 5, 7, 9, 11 and 13 executed against gravity. **(c)** Segments (Towards Gravity). Segments 6, 8, 10 and 12 executed towards gravity.

In addition the segments also differed in terms of the movement across the body i.e., from one side of the participant’s body towards the other side. The movement across the body is referred to as 'cross-body component’ through the rest of this article. Observations by the experimenter during the study showed that the segments that involved larger magnitude of cross-body component were perceived difficult to execute when compared to segments with smaller magnitude of cross-body component by majority of the participants. Therefore, alongside the first three conditions listed in Table 4, a fourth condition describing the cross-body component involved during every segment was also included in the data. Table 4 lists the lengths of all the segments and various conditions imposed during these segments. Figures 7(a)–(c) provide a pictorial representation of the thirteen segments, Figure 7(a) shows the segments at 'ground level’ , Figure 7(b) shows the segments that were 'against gravity’ and Figure 7(c) shows the segments that were 'towards gravity’. In these figures the 'reaching’ segments were identified by *red dotted lines* and the 'returning’ segments by *blue lines*.

**Table 4:** Segment details

***Segment***	***Length (m)***	***Condition-I***	***Condition-II***	***Condition-III***	***Condition-IV***
		***Embedded-Virtual***	***Reach-Return***	***Movement direction***	***Cross-body component***
Segment-1	0.350	Embedded	Reach	Ground-level	Large
Segment-2	0.320	Embedded	Return	Ground-level	Small
Segment-3	0.320	Virtual	Reach	Ground-level	Small
Segment-4	0.350	Virtual	Return	Ground-level	Large
Segment-5	0.415	Embedded	Reach	Against Gravity	Large
Segment-6	0.400	Embedded	Return	Towards Gravity	Small
Segment-7	0.400	Embedded	Reach	Against Gravity	Small
Segment-8	0.415	Embedded	Return	Towards Gravity	Large
Segment-9	0.276	Virtual	Reach	Against Gravity	Large
Segment-10	0.415	Virtual	Reach	Towards Gravity	Large
Segment-11	0.400	Virtual	Return	Against Gravity	Small
Segment-12	0.400	Embedded	Reach	Towards Gravity	Small
Segment-13	0.415	Virtual	Return	Against Gravity	Large

In order to facilitate the comparison of performance of the participants during different segments, the duration to execute a unit length of a segment was calculated as below and it was termed as 'Normalised duration’.

$$\begin{array}{c} \mathrm{Point}\;A\;\left(\mathrm{source}\right):\left(A_{x},A_{y},A_{z}\right)\;\\ \mathrm{Point}\;B\;\left(\mathrm{target}\right):\left(B_{x},B_{y},B_{z}\right)\\ {\mathrm{Segment}}_{\mathrm{magnitude}}=\sqrt{{\left(B_{x}-A_{x}\right)}^{2}+{\left(B_{y}-A_{y}\right)}^{2}+{\left(B_{z}-A_{z}\right)}^{2}}\\ \mathrm{Seg}\;\mathrm{Duratio}n_{\mathrm{Normalised}}=\frac{\mathrm{Seg}\;\mathrm{Duratio}n_{\mathrm{Recorded}}}{{\mathrm{Segment}}_{\mathrm{magnitude}}} \end{array}$$

The study is aware that different patterns for arm movements might include activities of different muscle groups and different muscle synergies. In our current experiment, we have chosen to investigate the lagging and leading attributes of movements in spite of this variation. A second study currently in preparation, investigates the influences caused by the activities of different muscles during performing segments shown by Table 4.

### Multivariate regression analysis

Our secondary goal was to study the variations in duration to execute a segment based on the set of conditions imposed to execute a segment. Hence regression was chosen as a suitable model, with duration to execute a segment as the outcome variable and set of conditions as predictors [21]. Similar analysis was performed previously on the data from the GENTLE/S clinical trials using multiple linear regression [22]. The regression was carried out using IBM SPSS 21.

The nature of various segments executed during the experiment differed in four conditions, Condition-I (Embedded-Virtual), Condition-II (Reach-Return), Con-dition-III (Against Gravity-Towards Gravity-Ground level), Condition-IV (Small-Large cross-body component). Table 5 provides the details of the categories under all the four conditions that are used as dummy (or predictor) variables in the regression. In order to study if the outcome variable (duration) of the regression model was considerably influenced by any of the participants, participants were also introduced as predictors into the regression model. The process detailed in [23] was used as reference in creating the dummy variables for the regression model.

**Table 5:** **Categories under four conditions *(reference category in bold)***

***Condition***	***Categories (Symbol)***
Condition I	**Virtual (EV0)**
	Embedded (EV1)
Condition II	**Return (RR0)**
	Reach (RR1)
Condition III	**Against Gravity (G0)**
	Towards Gravity (G1)
	Ground Level (G2)
Condition IV	**Large cross-body component (CB0)**
	Small cross-body component (CB1)

#### Model 1

The categories under all four conditions were keyed in as predictors into the regression model. Similarly participants were also included as predictors. Participant 1 was considered as reference participant in the regression model, as Participant 1 had greatest number of segments (11 out of 13) reaching to a constant optimum duration within the five iterations of the AA2 mode among all the participants of the study. The reference categories and the outcome variable (duration) remained the same for all the regression analysis models.

#### Model 2

Model 1 assumed that various conditions imposed during a segment independently influenced the performance of the participant. In order to investigate whether the influence of various conditions was mutually exclusive or had a combined effect on the performance of the participant interaction variables were created (as explained in [23]). Regression was run the second time with interaction variables included as additional predictor variables. The results from the first two regression models are reported in Tables 6, 7 and 8.

**Table 6:** Descriptive Statistics for dependent variable

***Dependent variable***	***Mean***	***Std. deviation***	***N***
Duration	8.582	2.706	2432

N - Number of cases.

**Table 7:** Model Summary

***Model***	***R***	***R square***	***Adjusted R square***	***Std. error of the estimate***	***Change statistics***					***Durbin-Watson***
					***R square change***	***F change***	***df1***	***df2***	***Sig. F change***	
Model 1	.737	.544	.537	1.84106	.544	84.021	34	2397	.000	
Model 2	.807	.651	.645	1.61329	.107	104.518	7	2390	.000	.957

**Table 8:** Coefficients from regression models 1 and 2

**Model**		**Unstandardized coefficients**		**Standardized coefficients**	**t**	**Sig.**
		**B**	**Std. Error**	**Beta**		
Model 1	(Constant)	7.457	.222		33.617	.000
	Embedded	-.650	.081	-.120	-8.027	.000
	Reach	.851	.076	.157	11.214	.000
	Towards Gravity	.399	.095	.068	4.206	.000
	Ground Level	.920	.091	.157	10.091	.000
	Small	-.151	.078	-.028	-1.950	.051
	Subject 2	-1.563	.276	-.117	-5.669	.000
	Participant 3	3.003	.284	.211	10.572	.000
	Participant 4	1.471	.284	.103	5.178	.000
	Participant 5	.838	.309	.050	2.710	.007
	Participant 6	4.903	.295	.319	16.630	.000
	Participant 7	2.891	.295	.188	9.805	.000
	Participant 8	-.390	.284	-.027	-1.374	.170
	Participant 9	1.073	.295	.070	3.640	.000
	Participant 10	.566	.295	.037	1.921	.055
	Participant 11	-.662	.284	-.046	-2.329	.020
	Participant 12	2.362	.295	.154	8.013	.000
	Participant 13	5.587	.295	.364	18.950	.000
	Participant 14	-1.445	.284	-.101	-5.087	.000
	Participant 15	-.340	.295	-.022	-1.154	.249
	Participant 16	-.379	.295	-.025	-1.285	.199
	Participant 17	.748	.295	.049	2.536	.011
	Participant 18	-.597	.295	-.039	-2.024	.043
	Participant 19	-1.449	.295	-.094	-4.914	.000
	Participant 20	2.323	.295	.151	7.879	.000
	Participant 21	-2.114	.295	-.138	-7.171	.000
	Participant 22	2.230	.295	.145	7.564	.000
	Participant 23	2.091	.284	.147	7.362	.000
	Participant 24	.264	.295	.017	.895	.371
	Participant 25	-1.835	.295	-.119	-6.224	.000
	Participant 26	.818	.295	.053	2.775	.006
	Participant 27	-.930	.295	-.061	-3.154	.002
	Participant 30	-.826	.295	-.054	-2.801	.005
	Participant 31	2.682	.295	.175	9.099	.000
	Participant 32	-.079	.294	-.005	-.269	.788
Model 2	(Constant)	6.358	.215		29.597	.000
	Embedded	-3.477	.167	-.641	-20.841	.000
	Reach	4.433	.167	.817	26.606	.000
	Towards Gravity	.117	.289	.020	.404	.686
	Ground Level	1.831	.167	.312	10.991	.000
	Small	.729	.167	.134	4.377	.000
	Participant 2	-1.563	.242	-.117	-6.470	.000
	Participant 3	3.003	.249	.211	12.065	.000
	Participant 4	1.471	.249	.103	5.909	.000
	Participant 5	.838	.271	.050	3.092	.002
	Participant 6	4.903	.258	.319	18.978	.000
	Participant 7	2.891	.258	.188	11.190	.000
	Participant 8	-.390	.249	-.027	-1.568	.117
	Participant 9	1.073	.258	.070	4.153	.000
	Participant 10	.566	.258	.037	2.192	.028
	Participant 11	-.662	.249	-.046	-2.658	.008
	Participant 12	2.362	.258	.154	9.145	.000
	Participant 13	5.587	.258	.364	21.626	.000
	Participant 14	-1.445	.249	-.101	-5.805	.000
	Participant 15	-.340	.258	-.022	-1.316	.188
	Participant 16	-.379	.258	-.025	-1.467	.143
	Participant 17	.748	.258	.049	2.894	.004
	Participant 18	-.597	.258	-.039	-2.309	.021
	Participant 19	-1.449	.258	-.094	-5.608	.000
	Participant 20	2.323	.258	.151	8.991	.000
	Participant 21	-2.114	.258	-.138	-8.184	.000
	Participant 22	2.230	.258	.145	8.632	.000
	Participant 23	2.091	.249	.147	8.402	.000
	Participant 24	.264	.258	.017	1.022	.307
	Participant 25	-1.835	.258	-.119	-7.103	.000
	Participant 26	.818	.258	.053	3.167	.002
	Participant 27	-.930	.258	-.061	-3.600	.000
	Participant 30	-.826	.258	-.054	-3.197	.001
	Participant 31	2.682	.258	.175	10.384	.000
	Participant 32	-.065	.258	-.004	-.253	.800
	EV1G1	4.193	.334	.653	12.569	.000
	EV1G2	2.831	.264	.377	10.732	.000
	RR1G1	-2.682	.289	-.358	-9.280	.000
	RR1G2	-3.759	.204	-.501	-18.401	.000
	RR1CB1	-1.361	.236	-.212	-5.770	.000
	G1CB1	-.390	.236	-.052	-1.653	.099
	G2CB1	.345	.167	.046	2.068	.039

### R square (R^2^) and adjusted R square

R^2^ (=0.651 for Model 2) implies that 65.1% variability in the outcome of dependent variable is accounted for by the dummy variables (predictors) that are included in the regression model. The regression model is considered as a good fit if the adjusted R^2^ is approximately equal to the R^2^. Considering the values of both R^2^ and Adjusted R^2^, Model 2 was a better fit for the data collected during this experiment when compared to Model 1.

### Change statistics

Change statistics explain the difference made by additional predictors to the regression model. Change statistics from Table 7 indicate that new predictors included in Model 2 made a significant (p <0.001) difference to the Model 1. The change statistics also indicate that Model 2 (with interaction variables), was a better fit of the data when compared to Model 1.

### Model parameters

Multiple linear regression can be represented in an equation form as shown below

(1) $$\begin{array}{ll} \mathrm{Dependent}\;\mathrm{variable}= & b_{0}+b_{1}\left({\mathrm{predictor}}_{1}\right)\\ & +b_{2}\left(\mathrm{predicto}r_{2}\right)+\ldots \\ & +b_{i}\left(\mathrm{predicto}r_{i}\right) \end{array}$$

The dependent variable for both Model 1 and Model 2 was 'duration’ to execute a segment and the predictors were the variables (except the 'Constant’) listed against Model 1 and Model 2 in Table 8. The coefficients listed in the column B (unstandardized coefficients) correspond to the *b*_*i*_ values for corresponding predictor variable. The greater the value of *b*_*i*_, the greater is the influence of the predictor on the regression model. In Model 1 (excluding the constant, *b*_0_ from Table 8), Participants 3, 6 and 13 had the highest *b*_*i*_ values. Model parameters therefore indicated that Participants 3, 6 and 13 could have influenced the regression model.

The *t*-test statistics for the predictors (Participants 3, 6 and 13)- listed under column 't’ in Model 1 (Table 8) were high as well as significant. Therefore both the *b*_*i*_ values and *t*-test statistics suggest that these three participants were making significant contribution towards the regression model. This could introduce a potential bias based on strength of these contributions.

#### Model 3 and Model 4

**Figure 8:**
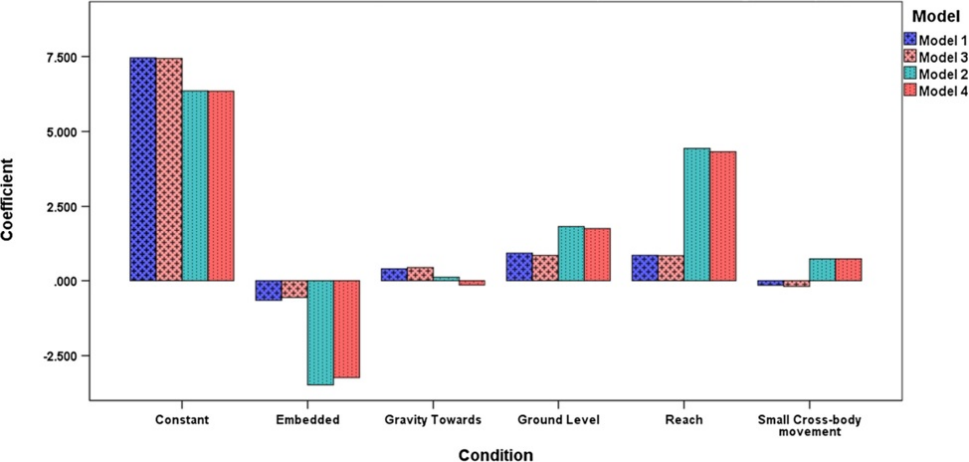
**Condition vs. Coefficient (Models 1-4).** Model 1 & Model 3 did not include interaction variables and Model 2 & Model 4 included interaction variables. The first two regression models used unfiltered data and the latter two used filtered data.

To avoid the potential bias, the data from these participants was excluded, and regression models 3 and 4 (similar to Model 1 and Model 2 respectively) were executed. Figure 8 shows a comparison of model parameters obtained from all four regression models. The comparison of bars from unfiltered data (Model 1 and Model 2) with bars from filtered data (Model 3 and Model 4) respectively shows that the regression models did not depend on the influencing participants. Figure 8 also shows that the regression models with interaction variables clearly differed from the regression models without interaction variables. This was also evident from the change statistics presented earlier in this section.

### Further analysis of interaction effects

Considering the *b*_*i*_ values of interaction variables from Model 2 that were significant (refer to the bottom rows of Table 8), it was evident that segments with help from an embedded object and the reaching segments were influenced by the direction of movement with respect to gravity. Substituting these *b*_*i*_ values from Table 8 into Equation 1, the durations for various combinations of conditions were calculated and presented in Table 9. A sample calculation (highlighted in bold in Table 9) with a combination of two out of four conditions listed in Table 5 is shown below.

**Table 9:** **Durations (sec) calculated by substituting *b***_***i ***_**values of interaction variables into Equation 1**

	**Against gravity (G0)**	**Towards gravity (G1)**	**Ground level (G2)**
**Virtual (EV0)**	6.358	6.475	8.190
**Embedded(EV1)**	2.881	**7.191**	7.543
**Return (RR0)**	6.358	6.475	8.190
**Reach (RR1)**	10.792	8.227	8.864

$$\begin{array}{ll} \mathrm{Duration} & =b_{0}+b_{\mathrm{Embedded}\;\left(\mathrm{EV}1\right)}+b_{\mathrm{Towards}\;\mathrm{Gravity}\;\left(G1\right)}\\ & \;+b_{\mathrm{Embedded}-\mathrm{Towards}\;\mathrm{Gravity}\;\left(\mathrm{EV}1G1\right)}\\ & =6.358+\left(-3.477\right)+0.117+4.193\\ & =7.191\;s \end{array}$$

The difference in execution times of the embedded and the virtual segments (Table 9), was greater when the movement was against gravity. In the other two cases (towards gravity and ground level) there existed a difference in the execution times of the virtual and the embedded segments, but the magnitude of the difference was less. In the case of reaching/returning segments, the reaching segments in general required longer execution times when compared to the returning segments, but the magnitude by which the execution times were longer depended on the direction of the movement with respect to gravity. There was also a mild interaction between the reaching and the cross-body component involved in executing the segment. The reaching segments with a large cross-body component required slightly longer execution times when compared to the reaching segments with small cross-body component. This difference was very small in the case of the returning segments.

## Discussion

The primary aim of this study was to test the adaptability of the GENTLE/A system, to the duration to execute point-to-point movements, according to the performance of the participant. The next aim was to study the influence of various conditions imposed during point-to-point movements on the performance of the participant.

The results from iteration level analysis showed that the algorithm could successfully tune the duration to the participant optimum constant value for point-to-point movements. Segment level analysis identified varying number of participants reaching a constant optimum value of duration during different segments. Furthermore it was observed that the default duration set at the beginning of the AA2 mode repetitions almost always scaled down during all the segments for all the participants by the end of the five repetitions. The differences in the performance identified at the iteration level and segment level inform that the number of repetitions required in reaching a constant optimum value of duration for various segments needs to be personalised.

Investigations into underlying reasons for the varying patterns of duration adaptation for different segments led to the study of the influence of the conditions imposed during different segments. Regression was chosen to carry out these investigations and among the four regression models, the models which included interaction variables (variables representing the interaction effects between various conditions) showed a better fit of data with greater R^2^ values.

### Embedded vs. Virtual

The results from the regression showed that segments with the target point represented by an embedded object required a shorter time for execution when compared to segments with the target point just displayed in the virtual environment. This result is consistent with a related study carried out by the colleagues in our research group [20]. The improved performance of healthy participants in the presence of a real-world object when compared to a virtual object clearly indicated that the virtual worlds are relatively tougher even for participants with good cognitive abilities. Considering the stroke patients with impaired cognitive abilities, an embedded set-up would be cognitively less demanding when compared to a complete virtual environment and might encourage and assist the participant in performing better during a therapy session. The future work with the GENTLE/A system also involves creating a complete embedded set-up to be tested in clinical settings.

### Reach vs. Return

In our previous studies with the GENTLE/A rehabilitation system, a constant duration was given to execute any segment and the reaching segments were always against gravity and the returning segments were always towards gravity. Results from a previous study demonstrated that participants failed to lead the robot most of the times during the returning segments when compared to the reaching movements [15]. The regression results from the current study showed that segments involving a reaching movement required longer execution time when compared to a returning movement irrespective of the gravity. One possible explanation for the difference in execution times could be the varying muscle groups involved to execute the reaching and the returning movements. This involves studying the kinematics of upper arm movements which forms part of our future work and publication is currently under preparation.

### Movement direction with respect to Gravity

When compared to embedded vs. virtual and reach vs. return, the different directions of movement with respect to gravity had a smaller influence on the duration to execute a point-to-point movement. Unexpectedly, the final durations after the five iterations of the AA2 mode for ground level segments were slightly longer when compared to segments with either gravity against or towards the direction of movement. Also the number of participants reaching a constant optimum value within five iterations for the ground level segments was relatively lower when compared to other segments with larger influence of gravity. A possible explanation could be that the segments that were executed at ground level were smaller in length when compared to other segments. The segments which are shorter in length would require less time for execution and hence the algorithm used for adapting the duration requires larger number of iterations to reach a constant optimum value, given that all segments start at same set duration. This point needs further consideration in our future work on improving our adaptation algorithm.

### Interaction effects

The conditions imposed during various segments not only influenced independently but also had interaction effects on the performance of the participant. These interaction effects were evident in Model 2 and Model 4. Segments with help from the embedded object were quicker to execute when compared to the virtual segments. This difference in execution times between the embedded and the virtual was largest when moving against gravity when compared to moving towards gravity or ground level movements. Similarly durations for the reaching segments when compared to the returning segments were longer when working against gravity and shorter when working towards gravity or at ground level. The cross-body component had a slightly greater impact on the reaching segments when compared to the returning segments. The influence of these interaction effects on the performance of the participant needs further investigation.

### Limitations and Future work

The embedded set-up implemented during this study had embedded objects placed closer to the actual target point in order to avoid collision of the participant’s arm with the embedded object. The embedded help offered was not uniform i.e., for some of the target points the embedded help was presented as Ping-Pong balls hung closer to the actual target point and for a couple of target points the embedded help was presented as a coloured sticker on the table-mat, just below the actual target point (refer Figure 3). It was evident from the results that the embedded segments were easier to execute when compared to the virtual segments in terms of duration, future work with the GENTLE/A system will develop a complete embedded set-up where similar targets can be executed in both the embedded and the virtual worlds and further comparisons can be made.

The influence of cross-body component was a new observation during the experiment. The influence of this condition was lower than expected from the experimenter’s observations. Cross-body component seems to show some influence on the reaching segments which requires further investigations based on involvement of different muscles in supporting different arm actions.

The influence of gravity as a stand-alone condition had a lower impact on the performance of the participants, when compared to other conditions. It was evident that the direction of movement with respect to gravity did have a small impact that was significant in combination with other conditions when interaction variables were introduced into the regression model. Comparing the data from movements executed under the influence of gravity with gravity compensated movements would give a better picture about the influence of gravity. These investigations form part of future studies with the GENTLE/A system. It is also notable that gravity effects on healthy participants could not be easily transferred to similar experiences with patients recovering from stroke, for instance, weakness and lack of coordination in stroke patients can provide a different set of observations.

## Conclusions

Our recent study with the GENTLE/A rehabilitation system, that can offer adaptive robotic assistance in upper limb rehabilitation, was presented in this paper. This study could successfully evaluate the adaptive nature of the GENTLE/A rehabilitation system with healthy subjects. The system could adjust itself and reach a constant optimum value of duration for a point-to-point movement using the contribution of the participants. Whether the adaptability of the GENTLE/A system would be of greater use in clinical settings, where a large variability is expected in the performance of the patients, is subject of our future research. However, this study shows that different patterns of arm movement, as well as different presentation for targets, can influence the durations set to achieve targets. This is an important consideration for studies applying a set duration to achieve reaching and returning trajectories. The constant optimum value for duration to which the system adjusts could also be used as an assessment parameter across the block of interaction sessions in clinical settings. The results from the study also showed that participants were quicker in executing point-to-point movements in the embedded set-up when compared to the virtual environment. This indicated that the embedded targets were better perceived when compared to the virtual targets shown on the monitor. The difference may be comparable or more pronounced in the case of stroke patients with comparatively lower cognitive abilities, so the use of the embedded and the real objects could be potentially less cognitively challenging for stroke patients. The reaching movements required longer execution times when compared to the returning movements irrespective of the influence of the gravity. Further investigations into the kinematics of the upper arm involved in the reaching and the returning movements might shed further light on the differences observed. The authors are therefore studying the physiology of the upper arm, muscle groups involved and their influence on various movements executed during this study and would intend to report the findings in their next publication.

## Competing interests

The authors declare that they have no competing interests.

## Authors’ contributions

The design of the study was agreed by RC and FA. RC recruited the participants, conducted the experiment and acquired the data. RC and FA were involved in analysing the data and interpreting the results. RC drafted the manuscript with critical revision of the manuscript from time to time by FA and KD. All the authors read and approved the final manuscript.

## Supplementary Material

Additional file 1: Table A Adaptation of segment durations (seconds) during the five iterations of the AA2 mode for all the participants. Click here for file
